# Poly[bis­[μ-1,4-bis­(1,2,4-triazol-1-ylmeth­yl)benzene-κ^2^ *N* ^4^:*N* ^4′^]dichloridomanganese(II)]

**DOI:** 10.1107/S1600536810026322

**Published:** 2010-07-14

**Authors:** Bin Ding, Hong-Ai Zou

**Affiliations:** a Tianjin Key Laboratory of Structure and Performance for Functional Molecules, Tianjin Normal University, Tianjin 300071, People’s Republic of China

## Abstract

The Mn^II^ atom in the title coordination polymer, [MnCl_2_(C_12_H_12_N_6_)_2_]_*n*_, lies on a center of inversion in a six-coordinate octa­hedral environment comprising four N-atom donors from four *N*-heterocyclic ligands and two chloride atoms. Bridging by the ligands results in a layer structure of a 14.79 (5) × 14.79 (5) Å (4,4) rhombic net topology, with the Mn^II^ atoms all lying on a plane. The parallel layers stack in an *ABCABC*… manner through inter­layer C—H⋯N and C—H⋯Cl hydrogen bonds.

## Related literature

For the preparation of highly stable, infinite metal–ligand frameworks by hydro­thermal methods, see: Chui *et al.* (1999 ▶); Gerrard & Wood (2000 ▶); Gutschke *et al.* (1996 ▶). For a three-dimensional self-catenating network involving the 1,4-bis­(triazol-1-ylmeth­yl)benzene ligand (*L*) which contains two different types of layers, see: Li *et al.* (2005 ▶). For a manganese inorganic–organic hybrid compound cont­ain­ing the flexible *L* ligand, [Mn_2_(H_2_O)_4_(*L*)_3_][SiMo_12_O_40_]·4H_2_O, see: Dong & Xu (2009 ▶).
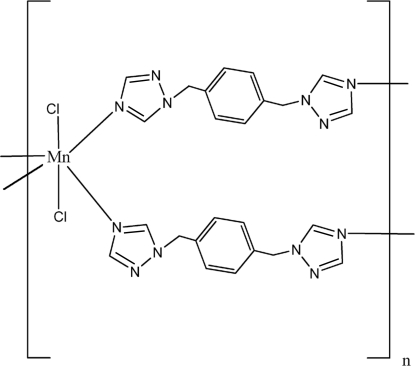


## Experimental

### 

#### Crystal data

[MnCl_2_(C_12_H_12_N_6_)_2_]
*M* *_r_* = 606.39
Monoclinic, 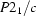

*a* = 7.5863 (16) Å
*b* = 21.925 (5) Å
*c* = 8.8442 (18) Å
β = 108.775 (4)°
*V* = 1392.8 (5) Å^3^
*Z* = 2
Mo *K*α radiation
μ = 0.70 mm^−1^
*T* = 294 K
0.20 × 0.14 × 0.08 mm

#### Data collection

Bruker APEXII CCD area-detector diffractometer
Absorption correction: multi-scan (*SADABS*; Sheldrick, 1996 ▶) *T*_min_ = 0.645, *T*_max_ = 1.000
7263 measured reflections
2587 independent reflections
1660 reflections with *I* > 2σ(*I*)
*R*_int_ = 0.050

#### Refinement

*R*[*F* ^2^ > 2σ(*F* ^2^)] = 0.040
*wR*(*F* ^2^) = 0.090
*S* = 1.00
2587 reflections
178 parameters
H-atom parameters constrained
Δρ_max_ = 0.37 e Å^−3^
Δρ_min_ = −0.27 e Å^−3^

### 

Data collection: *APEX2* (Bruker, 2007 ▶); cell refinement: *SAINT* (Bruker, 2007 ▶); data reduction: *SAINT*; program(s) used to solve structure: *SHELXS97* (Sheldrick, 2008 ▶); program(s) used to refine structure: *SHELXL97* (Sheldrick, 2008 ▶); molecular graphics: *SHELXTL* (Sheldrick, 2008 ▶) and *DIAMOND* (Brandenburg & Berndt, 2005 ▶); software used to prepare material for publication: *SHELXTL*.

## Supplementary Material

Crystal structure: contains datablocks global, I. DOI: 10.1107/S1600536810026322/ng2796sup1.cif

Structure factors: contains datablocks I. DOI: 10.1107/S1600536810026322/ng2796Isup2.hkl

Additional supplementary materials:  crystallographic information; 3D view; checkCIF report

## Figures and Tables

**Table 1:** Hydrogen-bond geometry (Å, °)

*D*—H⋯*A*	*D*—H	H⋯*A*	*D*⋯*A*	*D*—H⋯*A*
C9—H9⋯N5^i^	0.93	2.61	3.523 (4)	168
C11—H11⋯Cl1^ii^	0.93	2.77	3.635 (3)	155

Symmetry codes: (i) 
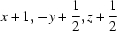
; (ii) 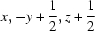.

**Fig. 1.**
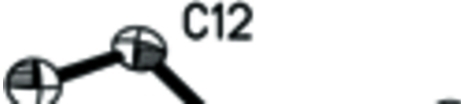
A view of the local coordination of the MnII cations in (I), showing the atom-numbering scheme. Displacement ellipsoids are drawn at the 30% probability level. [Symmetry codes: (i) 1 + x,-1/2 - y,-1/2 + z; (ii) -x,1/2 + y, 1/2 - z; (iii) 1 - x, 1 - y, -z.]

**Fig. 2.**
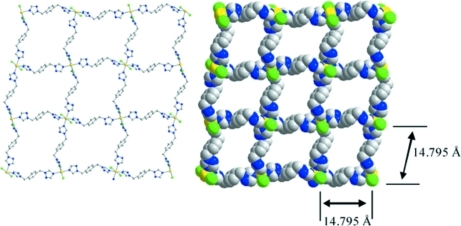
Schematic representation of the (4,4) network topology of (I) viewed down the c axis.

**Fig. 3.**
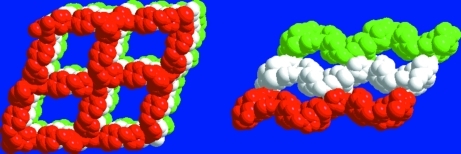
Top (a) and side (b) views showing the packing of the grid layers in (I).

## supplementary crystallographic information

### Comment

The design and synthesis of highly-dimensional metal-organic framework (MOF) structures is becoming an increasing popular field of research in view of the formation of fascinating structures and their potentially useful ion-exchange, adsorption, catalytic, fluorescence, and magnetic properties. Triazole-containing ligands, such as 1,4-bis(triazol-1-ylmethyl)benzene, 1,2-bis(1,2,4-triazole-1-yl)ethane, 1,2-bis(1,2,4-triazole-1-yl)hexane and related species, represent another class of N-donor organic linkers for constructing coordination polymers, and these ligands have been proved to be able to produce architectures different from those obtained from pyridyl-based ligands.With two methylene *sp*^3^-carbon atoms, the ligand 1,4-bis(triazol-1-ylmethyl)benzene *L* is highly flexible and can assume variable *trans* and *gauche* conformations. It can generate unusual 2-D polyrotaxane networks, 3-D entangled networks and 3-D self-catenating network containing two different types of layers (Li *et al.*, 2005). However, to date, only one manganese hybrid compound [Mn_2_(H_2_O)_4_(*L*)_3_][SiMo_12_O_40_].4H_2_O with the flexible *L* ligands has been reported in the present of molybdate recently (Dong *et al.*, 2009).

Several synthetic methods can be used to obtain new polymeric complexes. In the previous literatures the hydrothermal method has been a promising technique in preparing highly stable, infinite metal–ligand frameworks (Gutschke *et al.*, 1996; Chui *et al.*, 1999; Gerrard *et al.*, 2000). Taking the advantage of bridging ability of *L* in the chemical design of metal-organic molecular assemblies, under hydrothermal condition, we synthesized a novel 2-D (4,4) square grid layer manganese(II) coordination polymer, poly-[[bis[µ_2_-1,4-bis(triazol-1-ylmethyl)benzene-bis(chloride)manganese(II)].

Selected bond distances and bond angles are listed in Table 1. Fig. 1 shows the coordination environment of the metal ions in 1. The asymmetric unit of (I) contains one Mn^II^ cation, four-half of *L* ligands and two chloride atoms. The Mn(II) ion, which resides at an inversion center, is octahedrally coordinated by two pairs of equivalent imine nitrogen atoms from *L* ligands in the equatorial plane (N1, N6^i^, N6^ii^, N1^iii^) and two equivalent terminal chloride ions occupying the axial positions (Cl1 and Cl1^iii^). Two triazole rings of the ligand rotate along the C—C single bond axis with the dihedral of 52.1°. The benzene groups have different twists relative to their adjacent triazole ring with the dihedral of 70.2° and 71.0°, respectively. The *cis* N–Mn–N bond angles range from 87.4 (8)° to 92.5 (2)°, and the axial Mn–Cl(chloride) distances (2.5666 (5) Å) are much longer than the equatorial Mn–*N*(imine) distances (2.251 (3) and 2.268 (3) Å), indicative of the distorted octahedral environment with a slight axial elongated. Each Mn(II) ion is linked by four equivalent *L* ligands in the *trans-* conformation to its four neighboring Mn(II) ions, thus affording 2-D (4,4) grid layers parallel to the crystallographic *ab* plane (Fig. 2). All the Mn(II) ions in each layer are strictly coplanar.

The grid motif has the dimensions of 14.79 (5) Å × 14.79 (5) Å (metal- to-metal distances), and the Mn···Mn···Mn corner angles (84.3 (7)° and 95.6 (3)°) within the motif are close to 90°, suggesting a rhombic geometry. The grid layers are closely stacked in an offset way (Fig. 3), with the cavity of each layer being occupied by the groups from the two neighboring layers, which are generated from the original one by unit translations along the a direction. Due to the interdigitation between neighboring layers. The nearest interlayer Mn···Mn distances are 7.568 (2) and 8.844 (2)Å between the neighboring layers [Mn1···Mn1(*x* + 1, *y*, *z*)] and between the next-nearest neighboring layers [Mn1···Mn1(*x*, *y*, *z* - 1)], respectively, both being much shorter than the intralayer one. Further inspection into the structure revealed that there exist interlayer weak C–H···N and C–H···Cl hydrogen bonds. The uncoordinated *L* nitrogen atoms and chloride ions of one layer, forms the hydrogen bonds with a triazole C–H groups from different *L* ligands of the neighboring layer: C(9)–H(9)···N(5) (1 + *x*,1/2 - *y*,1/2 + *z*) and C(11)–H(11)···Cl(1) (*x*,1/2 - *y*,1/2 + *z*). The C—H···N and C—H···Cl distances and the correpsonding angle are, respectively, 3.523 (4) Å and 168.0 (8)° for the former, and 3.635 (2) Å and 154.8 (5)° for the latter. The result also reveals that there is still great potential in the construction of those novel coordination polymers with highly flexible bis-triazole ligands.

### Experimental

A mixture of MnCl_2_.6H_2_O (0.2 mmol, 46.6 mg), *L* (0.4 mmol, 96.8 mg) and H_2_O (18.0 ml) in the molar radio of 1: 2:5000 was sealed in a 25 ml stainless steel reactor with Teflon liner and directly heated to 453 K ke pt at 453 K for 72 h, and then slowly cooled to 303 K at a rate for 4 K/h. Colorless block crystals of the title complex were collected by filtration and washed with ethanol (2 × 5 ml). Yield: 35%.

### Refinement

The H atoms of the aromatic rings were placed at calculated positions, with C—H = 0.93 Å. All H atoms were assigned fixed isotropic displacement parameters, with *U*_iso_(H) = 1.2Ueq(C) or 1.5Ueq(O).

### Crystal data

**Table:**

[MnCl_2_(C_12_H_12_N_6_)_2_]	*F*(000) = 622
*M**_r_* = 606.39	*D*_x_ = 1.446 Mg m^−^^3^
Monoclinic, *P*2_1_/*c*	Mo *K*α radiation, λ = 0.71073 Å
Hall symbol: -P 2ybc	Cell parameters from 1682 reflections
*a* = 7.5863 (16) Å	θ = 2.8–23.5°
*b* = 21.925 (5) Å	µ = 0.70 mm^−^^1^
*c* = 8.8442 (18) Å	*T* = 294 K
β = 108.775 (4)°	Block, colorless
*V* = 1392.8 (5) Å^3^	0.20 × 0.14 × 0.08 mm
*Z* = 2	

### Data collection

**Table:**

Bruker APEXII CCD area-detector diffractometer	2587 independent reflections
Radiation source: fine-focus sealed tube	1660 reflections with *I* > 2σ(*I*)
graphite	*R*_int_ = 0.050
phi and ω scans	θ_max_ = 25.5°, θ_min_ = 1.9°
Absorption correction: multi-scan (*SADABS*; Sheldrick, 1996)	*h* = −9→9
*T*_min_ = 0.645, *T*_max_ = 1.000	*k* = −19→26
7263 measured reflections	*l* = −7→10

### Refinement

**Table:**

Refinement on *F*^2^	Primary atom site location: structure-invariant direct methods
Least-squares matrix: full	Secondary atom site location: difference Fourier map
*R*[*F*^2^ > 2σ(*F*^2^)] = 0.040	Hydrogen site location: inferred from neighbouring sites
*wR*(*F*^2^) = 0.090	H-atom parameters constrained
*S* = 1.00	*w* = 1/[σ^2^(*F*_o_^2^) + (0.0412*P*)^2^] where *P* = (*F*_o_^2^ + 2*F*_c_^2^)/3
2587 reflections	(Δ/σ)_max_ < 0.001
178 parameters	Δρ_max_ = 0.37 e Å^−^^3^
0 restraints	Δρ_min_ = −0.27 e Å^−^^3^

### Special details

**Table:**

Geometry. All e.s.d.'s (except the e.s.d. in the dihedral angle between two l.s. planes) are estimated using the full covariance matrix. The cell e.s.d.'s are taken into account individually in the estimation of e.s.d.'s in distances, angles and torsion angles; correlations between e.s.d.'s in cell parameters are only used when they are defined by crystal symmetry. An approximate (isotropic) treatment of cell e.s.d.'s is used for estimating e.s.d.'s involving l.s. planes.
Refinement. Refinement of *F*^2^ against ALL reflections. The weighted *R*-factor *wR* and goodness of fit *S* are based on *F*^2^, conventional *R*-factors *R* are based on *F*, with *F* set to zero for negative *F*^2^. The threshold expression of *F*^2^ > σ(*F*^2^) is used only for calculating *R*-factors(gt) *etc*. and is not relevant to the choice of reflections for refinement. *R*-factors based on *F*^2^ are statistically about twice as large as those based on *F*, and *R*- factors based on ALL data will be even larger.

### Fractional atomic coordinates and isotropic or equivalent isotropic displacement parameters (Å^2^)

**Table:**

	*x*	*y*	*z*	*U*_iso_*/*U*_eq_	
Mn1	0.5000	0.5000	0.0000	0.03010 (18)	
Cl1	0.15989 (9)	0.47621 (4)	−0.16003 (8)	0.0404 (2)	
N1	0.4590 (3)	0.45414 (11)	0.2160 (3)	0.0350 (6)	
N2	0.5081 (3)	0.42190 (13)	0.4681 (3)	0.0549 (8)	
N3	0.3232 (3)	0.42456 (11)	0.3864 (3)	0.0339 (6)	
N4	−0.2711 (3)	0.14940 (11)	0.2963 (3)	0.0357 (6)	
N5	−0.4262 (3)	0.17945 (12)	0.2994 (3)	0.0462 (7)	
N6	−0.4061 (3)	0.08737 (11)	0.4162 (3)	0.0345 (6)	
C1	0.5808 (4)	0.44071 (16)	0.3598 (4)	0.0528 (9)	
H1	0.7087	0.4445	0.3815	0.063*	
C2	0.2973 (4)	0.44391 (13)	0.2394 (3)	0.0372 (7)	
H2	0.1814	0.4496	0.1626	0.045*	
C3	0.1824 (4)	0.40794 (15)	0.4602 (3)	0.0451 (8)	
H3A	0.0868	0.4391	0.4366	0.054*	
H3B	0.2401	0.4061	0.5752	0.054*	
C4	0.0941 (4)	0.34717 (14)	0.4007 (3)	0.0343 (7)	
C5	−0.0630 (4)	0.34410 (15)	0.2691 (4)	0.0493 (8)	
H5	−0.1170	0.3799	0.2191	0.059*	
C6	−0.1420 (4)	0.28890 (16)	0.2101 (4)	0.0528 (9)	
H6	−0.2486	0.2880	0.1210	0.063*	
C7	−0.0655 (4)	0.23505 (14)	0.2810 (4)	0.0379 (7)	
C8	0.0892 (4)	0.23780 (15)	0.4153 (4)	0.0462 (8)	
H8	0.1405	0.2020	0.4675	0.055*	
C9	0.1687 (4)	0.29315 (15)	0.4731 (4)	0.0450 (8)	
H9	0.2747	0.2941	0.5627	0.054*	
C10	−0.1494 (4)	0.17476 (15)	0.2130 (4)	0.0494 (9)	
H10A	−0.2205	0.1801	0.1010	0.059*	
H10B	−0.0501	0.1460	0.2195	0.059*	
C11	−0.2619 (4)	0.09541 (14)	0.3656 (3)	0.0363 (7)	
H11	−0.1671	0.0671	0.3771	0.044*	
C12	−0.5005 (4)	0.13984 (15)	0.3729 (3)	0.0420 (8)	
H12	−0.6111	0.1476	0.3935	0.050*	

### Atomic displacement parameters (Å^2^)

**Table:**

	*U*^11^	*U*^22^	*U*^33^	*U*^12^	*U*^13^	*U*^23^
Mn1	0.0284 (3)	0.0335 (4)	0.0321 (3)	0.0000 (3)	0.0150 (3)	0.0012 (3)
Cl1	0.0284 (4)	0.0517 (5)	0.0421 (4)	−0.0048 (3)	0.0130 (3)	−0.0030 (4)
N1	0.0309 (13)	0.0435 (17)	0.0337 (14)	−0.0009 (11)	0.0147 (11)	0.0074 (11)
N2	0.0436 (17)	0.072 (2)	0.0460 (17)	−0.0027 (14)	0.0098 (14)	0.0184 (14)
N3	0.0342 (14)	0.0362 (16)	0.0342 (14)	−0.0088 (11)	0.0150 (12)	0.0015 (11)
N4	0.0338 (14)	0.0381 (17)	0.0365 (14)	−0.0072 (12)	0.0131 (11)	−0.0015 (11)
N5	0.0372 (15)	0.0432 (19)	0.0556 (17)	0.0008 (13)	0.0112 (13)	0.0039 (13)
N6	0.0284 (13)	0.0368 (17)	0.0402 (14)	−0.0008 (11)	0.0139 (11)	0.0026 (11)
C1	0.0334 (18)	0.074 (3)	0.053 (2)	0.0024 (17)	0.0168 (17)	0.0196 (18)
C2	0.0343 (17)	0.045 (2)	0.0317 (17)	−0.0052 (14)	0.0096 (13)	0.0052 (14)
C3	0.053 (2)	0.050 (2)	0.0429 (18)	−0.0138 (16)	0.0298 (16)	−0.0023 (15)
C4	0.0370 (17)	0.041 (2)	0.0308 (17)	−0.0088 (14)	0.0194 (14)	0.0019 (13)
C5	0.056 (2)	0.040 (2)	0.046 (2)	−0.0011 (17)	0.0080 (17)	0.0124 (15)
C6	0.052 (2)	0.055 (3)	0.039 (2)	−0.0102 (18)	−0.0037 (16)	0.0065 (16)
C7	0.0440 (19)	0.035 (2)	0.0407 (18)	−0.0093 (15)	0.0226 (15)	−0.0005 (14)
C8	0.0414 (19)	0.038 (2)	0.055 (2)	0.0044 (16)	0.0105 (17)	0.0117 (16)
C9	0.0341 (18)	0.047 (2)	0.048 (2)	−0.0074 (16)	0.0036 (15)	0.0023 (16)
C10	0.061 (2)	0.050 (2)	0.047 (2)	−0.0185 (17)	0.0307 (17)	−0.0056 (16)
C11	0.0314 (16)	0.038 (2)	0.0403 (17)	0.0006 (14)	0.0122 (14)	0.0005 (14)
C12	0.0276 (16)	0.042 (2)	0.058 (2)	−0.0003 (15)	0.0167 (15)	−0.0029 (16)

### Geometric parameters (Å, °)

**Table:**

Mn1—N6^i^	2.251 (2)	C2—H2	0.9300
Mn1—N6^ii^	2.251 (2)	C3—C4	1.508 (4)
Mn1—N1^iii^	2.267 (2)	C3—H3A	0.9700
Mn1—N1	2.267 (2)	C3—H3B	0.9700
Mn1—Cl1^iii^	2.5655 (8)	C4—C5	1.373 (4)
Mn1—Cl1	2.5655 (8)	C4—C9	1.378 (4)
N1—C2	1.327 (3)	C5—C6	1.377 (4)
N1—C1	1.342 (3)	C5—H5	0.9300
N2—C1	1.316 (3)	C6—C7	1.375 (4)
N2—N3	1.357 (3)	C6—H6	0.9300
N3—C2	1.321 (3)	C7—C8	1.376 (4)
N3—C3	1.466 (3)	C7—C10	1.506 (4)
N4—C11	1.325 (3)	C8—C9	1.378 (4)
N4—N5	1.356 (3)	C8—H8	0.9300
N4—C10	1.464 (3)	C9—H9	0.9300
N5—C12	1.315 (4)	C10—H10A	0.9700
N6—C11	1.320 (3)	C10—H10B	0.9700
N6—C12	1.344 (4)	C11—H11	0.9300
N6—Mn1^iv^	2.251 (2)	C12—H12	0.9300
C1—H1	0.9300		
			
N6^i^—Mn1—N6^ii^	180.0	N3—C3—H3A	109.3
N6^i^—Mn1—N1^iii^	92.51 (8)	C4—C3—H3A	109.3
N6^ii^—Mn1—N1^iii^	87.49 (8)	N3—C3—H3B	109.3
N6^i^—Mn1—N1	87.49 (8)	C4—C3—H3B	109.3
N6^ii^—Mn1—N1	92.51 (8)	H3A—C3—H3B	107.9
N1^iii^—Mn1—N1	180.00 (12)	C5—C4—C9	117.8 (3)
N6^i^—Mn1—Cl1^iii^	90.81 (6)	C5—C4—C3	120.4 (3)
N6^ii^—Mn1—Cl1^iii^	89.19 (6)	C9—C4—C3	121.8 (3)
N1^iii^—Mn1—Cl1^iii^	89.34 (6)	C4—C5—C6	121.2 (3)
N1—Mn1—Cl1^iii^	90.66 (6)	C4—C5—H5	119.4
N6^i^—Mn1—Cl1	89.19 (6)	C6—C5—H5	119.4
N6^ii^—Mn1—Cl1	90.81 (6)	C7—C6—C5	120.9 (3)
N1^iii^—Mn1—Cl1	90.66 (6)	C7—C6—H6	119.6
N1—Mn1—Cl1	89.34 (6)	C5—C6—H6	119.6
Cl1^iii^—Mn1—Cl1	180.0	C6—C7—C8	118.3 (3)
C2—N1—C1	101.8 (2)	C6—C7—C10	120.6 (3)
C2—N1—Mn1	126.18 (18)	C8—C7—C10	121.1 (3)
C1—N1—Mn1	131.04 (18)	C7—C8—C9	120.6 (3)
C1—N2—N3	101.7 (2)	C7—C8—H8	119.7
C2—N3—N2	109.8 (2)	C9—C8—H8	119.7
C2—N3—C3	128.3 (2)	C8—C9—C4	121.3 (3)
N2—N3—C3	121.9 (2)	C8—C9—H9	119.4
C11—N4—N5	110.0 (2)	C4—C9—H9	119.4
C11—N4—C10	128.7 (2)	N4—C10—C7	112.7 (2)
N5—N4—C10	121.2 (2)	N4—C10—H10A	109.0
C12—N5—N4	101.6 (2)	C7—C10—H10A	109.0
C11—N6—C12	102.3 (2)	N4—C10—H10B	109.0
C11—N6—Mn1^iv^	127.6 (2)	C7—C10—H10B	109.0
C12—N6—Mn1^iv^	128.76 (18)	H10A—C10—H10B	107.8
N2—C1—N1	115.9 (3)	N6—C11—N4	110.5 (3)
N2—C1—H1	122.1	N6—C11—H11	124.8
N1—C1—H1	122.1	N4—C11—H11	124.8
N3—C2—N1	110.8 (2)	N5—C12—N6	115.6 (2)
N3—C2—H2	124.6	N5—C12—H12	122.2
N1—C2—H2	124.6	N6—C12—H12	122.2
N3—C3—C4	111.7 (2)		
			
N6^i^—Mn1—N1—C2	69.1 (2)	N3—C3—C4—C5	−90.4 (3)
N6^ii^—Mn1—N1—C2	−110.9 (2)	N3—C3—C4—C9	88.3 (3)
N1^iii^—Mn1—N1—C2	−133 (100)	C9—C4—C5—C6	−0.9 (4)
Cl1^iii^—Mn1—N1—C2	159.9 (2)	C3—C4—C5—C6	177.9 (3)
Cl1—Mn1—N1—C2	−20.1 (2)	C4—C5—C6—C7	−0.2 (5)
N6^i^—Mn1—N1—C1	−97.5 (3)	C5—C6—C7—C8	1.8 (5)
N6^ii^—Mn1—N1—C1	82.5 (3)	C5—C6—C7—C10	−178.3 (3)
N1^iii^—Mn1—N1—C1	60 (100)	C6—C7—C8—C9	−2.4 (4)
Cl1^iii^—Mn1—N1—C1	−6.7 (3)	C10—C7—C8—C9	177.7 (3)
Cl1—Mn1—N1—C1	173.3 (3)	C7—C8—C9—C4	1.3 (5)
C1—N2—N3—C2	0.2 (3)	C5—C4—C9—C8	0.3 (4)
C1—N2—N3—C3	−179.4 (3)	C3—C4—C9—C8	−178.5 (2)
C11—N4—N5—C12	0.0 (3)	C11—N4—C10—C7	−124.3 (3)
C10—N4—N5—C12	175.2 (2)	N5—N4—C10—C7	61.5 (4)
N3—N2—C1—N1	−1.0 (4)	C6—C7—C10—N4	−98.4 (3)
C2—N1—C1—N2	1.4 (4)	C8—C7—C10—N4	81.5 (4)
Mn1—N1—C1—N2	170.3 (2)	C12—N6—C11—N4	−0.1 (3)
N2—N3—C2—N1	0.7 (3)	Mn1^iv^—N6—C11—N4	167.33 (17)
C3—N3—C2—N1	−179.7 (3)	N5—N4—C11—N6	0.1 (3)
C1—N1—C2—N3	−1.2 (3)	C10—N4—C11—N6	−174.7 (2)
Mn1—N1—C2—N3	−170.87 (18)	N4—N5—C12—N6	−0.1 (3)
C2—N3—C3—C4	74.3 (4)	C11—N6—C12—N5	0.1 (3)
N2—N3—C3—C4	−106.2 (3)	Mn1^iv^—N6—C12—N5	−167.11 (19)

Symmetry codes: (i) −*x*, *y*+1/2, −*z*+1/2; (ii) *x*+1, −*y*+1/2, *z*−1/2; (iii) −*x*+1, −*y*+1, −*z*; (iv) −*x*, *y*−1/2, −*z*+1/2.

### Hydrogen-bond geometry (Å, °)

**Table:**

*D*—H···*A*	*D*—H	H···*A*	*D*···*A*	*D*—H···*A*
C9—H9···N5^v^	0.93	2.61	3.523 (4)	168
C11—H11···Cl1^vi^	0.93	2.77	3.635 (3)	155

Symmetry codes: (v) *x*+1, −*y*+1/2, *z*+1/2; (vi) *x*, −*y*+1/2, *z*+1/2.
